# Supplementary material for: Seroprevalence of anti-SARS-CoV-2 antibodies in Thai adults during the first three epidemic waves
Source: PLoS One. 2022 Apr 27;17(4):e0263316. doi: 10.1371/journal.pone.0263316 (PMC9045619; doi:10.1371/journal.pone.0263316)
Supplement: S2 Table — (DOCX) [file pone.0263316.s002.docx]

**S2 Table. Verification of SARS-CoV-2 seropositive cases using microNT, CLIA-Architect IgG, and IFA.**

| **Group** | **Number positive** | | |
| --- | --- | --- | --- |
|  | **microNT** | **CLIA-Architect IgG** | **Final result after IFA verification** |
| Patients | 122 | 115 | 124 |
|  | (11 discordant results) | |  |
| Entertainment workers | 3 | 4 | 3 |
|  | (7 discordant results) | |  |
| Participants who shared workplaces or communities | 38 | 27 | 38 |
|  | (11 discordant results) | |  |
| Thais who returned from abroad | 155 | 143 | 155 |
|  | (16 discordant results) | |  |
